# Aberrant CDKN1A transcriptional response associates with abnormal sensitivity to radiation treatment

**DOI:** 10.1038/sj.bjc.6604381

**Published:** 2008-05-20

**Authors:** C Badie, S Dziwura, C Raffy, T Tsigani, G Alsbeih, J Moody, P Finnon, E Levine, D Scott, S Bouffler

**Affiliations:** 1 Radiation Effects Department, Health Protection Agency, Centre for Radiation Chemical and Environmental Hazards, Radiation Protection Division, Chilton, Didcot, Oxfordshire OX11 ORQ, UK; 2 Radiation Biology Laboratory, Biomedical Physics Department, King Faisal Specialist Hospital & Research Centre, Riyadh 11211, Saudi Arabia; 3 Clinical Oncology Department, Christie Hospital NHS Foundation Trust, Wilmslow Road, Manchester M20 4BX, UK; 4 Cancer Genetics Department, Paterson Institute for Cancer Research, Christie Hospital NHS Foundation Trust, Manchester M20 4BX, UK

**Keywords:** gene expression, radiation therapy, normal tissue response, lymphocyte, QRT-PCR (quantitative real-time polymerase chain reaction), SNPs (single nucleotide polymorphisms)

## Abstract

Normal tissue reactions to radiation therapy vary in severity among patients and cannot be accurately predicted, limiting treatment doses. The existence of heritable radiosensitivity syndromes suggests that normal tissue reaction severity is determined, at least in part, by genetic factors and these may be revealed by differences in gene expression. To test this hypothesis, peripheral blood lymphocyte cultures from 22 breast cancer patients with either minimal (11) or very severe acute skin reactions (11) have been used to analyse gene expression. Basal and post-irradiation expression of four radiation-responsive genes (*CDKN1A*, *GADD45A*, *CCNB1*, and *BBC3*) was determined by quantitative real-time PCR in T-cell cultures established from the two patient groups before radiotherapy. Relative expression levels of *BBC3*, *CCNB1*, and *GADD45A* 2 h following 2 Gy X-rays did not discriminate between groups. However, post-irradiation expression response was significantly reduced for *CDKN1A* (*P*<0.002) in severe reactors compared to normal. Prediction of reaction severity of ∼91% of individuals sampled was achieved using this end point. Analysis of *TP53* Arg72Pro and *CDKN1A* Ser31Arg single nucleotide polymorphisms did not show any significant association with reaction sensitivity. Although these results require confirmation and extension, this study demonstrates the possibility of predicting the severity of acute skin radiation toxicity in simple tests.

Radiation therapy is used to treat ∼50% of all cancer patients. However, radiation therapy can cause a range of adverse normal tissue responses, which can limit therapeutic doses delivered. Early reactions principally affect high turn-over tissues, such as skin, gastrointestinal tract and bone marrow. During treatment or within a few weeks of completing a fractionated radiotherapy course, skin erythema, dry or moist desquamation of the skin, mucositis, nausea and diarrhoea are typical signs of radiation toxicity. Prediction of those patients at risk of severe reactions is difficult. Considerable efforts have been made to correlate normal tissue toxicity with cellular responses to ionising radiation (IR). However, no significant relationships have yet been found between fibroblast or lymphocyte cytotoxicity and acute (Geara *et al*, 1993; Brock *et al*, 1995; Johansen *et al*, 1996) or late (Russell *et al*, 1998; Peacock *et al*, 2000) normal tissue reactions to IR. However, normal cell radiosensitivity in some cases may be an important factor predictive of radiation therapy response as illustrated by West *et al* (2001) whose results showed that blood lymphocyte radiosensitivity (SF2) is a highly significant prognostic factor for the risk of developing late radiation morbidity. In terms of other end points, Barber *et al* (2000) evaluated the predictive value of lymphocyte chromosome radiosensitivity in patients receiving radiotherapy for breast cancer and concluded that these assays perform poorly in predicting normal tissue effects. Wang *et al* (2005) and Lopez *et al* (2005) both failed to find a correlation between DNA repair capacities in peripheral blood lymphocytes and acute skin reaction during radiotherapy. To date, no cellular or molecular assay has been used in clinics to predict the severity of radiotherapy reactions. It is not clear if this is due to the absence of a suitable assay or the lack of a suitable indicator cell/tissue.

It is likely that individual radiosensitivity has a heritable component as demonstrated in syndromes such as ataxia telangiectasia (AT), Nijmegen breakage syndrome and Fanconi's anaemia (Andreassen, 2005). Some evidence suggests that gene expression patterns are abnormal in radiosensitive conditions such as AT (Watts *et al*, 2002); heterozygosity for mutation in ATM (ataxia telangiectasia mutated) may occur in 1% of individuals and has been associated with sensitivity to IR *in vitro* (Smilenov *et al*, 2001). Furthermore, gene expression can be affected by radiation exposure and some of these responses have a heritable component (Correa and Cheung, 2004). It is, therefore, reasonable to postulate that genetic variation contributes to determining the severity of response by irradiated tissues in the body and that this variation is reflected in gene expression patterns. Rapid and simple predictive assays of radiation response in easily accessible cells, for example lymphocytes, would bring clinical benefits (lymphocytes are an ideal cell population as they can be obtained by a minimally invasive method combining simplicity and rapidity as well as reliability, also repeat sampling is easy). Using multigene classifiers, earlier work showed that lymphoblastoid cell lines from patients with acute radiation toxicity have abnormal transcriptional responses to radiation (Rieger *et al*, 2004). Svensson *et al* (2006) also reported that changes of expression in a specific set of genes after *in vitro* irradiation of stimulated peripheral lymphocytes can, to some extent, successfully predict severe late reaction status. Using subcutaneous fibroblasts from breast cancer patients, Rodningen *et al* (2007) identified a set of 18 radiation-responsive genes, which may provide a predictive assay for late normal tissue reactions after radiotherapy. The amount of information available on gene expression responses to radiation has been increasing considerably in recent years (Kruse and Stewart, 2007). These studies of altered gene expression have been useful for elucidating the molecular mechanisms underlying cellular radiation response and a few have been able to identify genes as potential indicators of severe reactions to radiotherapy treatment. Surprisingly, in these studies, the most pronounced radiation-responsive genes (Rieger and Chu, 2004), which show high variation in expression between individuals, do not seem to be predictive of radiation toxicity. Those, which have been identified as informative, are associated with various pathways and differ between studies thus complicating the interpretation of the data; this remains a challenge, particularly at the level of individual gene expression. Our approach was to re-examine, in a rigorous quantitative manner, the expression response of a fewer number of genes associated with relevant pathways and previously identified as radiation responsive, in an attempt to correlate expression levels with normal tissue reaction to IR.

Exposure of cells to IR induces a large range of DNA alterations and results in complex biological responses. The DNA-damage response (DDR) network mediates DNA repair, cell cycle checkpoints and/or apoptosis. In response to DNA double-strand breaks, the *ATM* gene with its regulator the MRN (*Mre11-Rad50-NBS1*) complex are activated (Lee and Paull, 2005). Ataxia telangiectasia mutated protein is auto-phosphorylated at Ser-1981 in response to DNA damage, which causes dissociation of the inactive dimers (Bakkenist and Kastan, 2003). Ataxia telangiectasia mutated kinase is essential for activation of cell cycle checkpoints and DNA repair in response to IR. It acts upstream of p53 by activating p53 protein through phosphorylation of Ser-15 (Banin *et al*, 1998; Canman *et al*, 1998) leading to p53 stabilisation.

p53 protein is also activated through a number of other post-translational modifications, including phosphorylations, acetylations and methylation (Toledo and Wahl, 2006). After DNA damage induced by IR, cells enter either cell-cycle arrest or apoptosis, depending on which of the pathways is predominant in the specific cell type and environment. p53 functions as a crucial transcription factor in a well established response through direct protein binding to target gene promoter elements. Although cell-cycle arrest depends on the ability of p53 to induce the transcription of genes such as *CDKN1A* and *GADD45*, apoptosis depends on induction of a distinct class of genes including *BBC3* also called *PUMA* (p53 upregulated modulator of apoptosis) (Yu *et al*, 2003). p53 also negatively regulates the expression of cell-cycle regulator genes such as *CYCB1* (Badie *et al*, 2000; Imbriano *et al*, 2005). In this study, we speculated that patients overreacting to radiotherapy treatment may have abnormal transcriptional responses in one or more of the genes involved specifically in DDR pathways. Among a wide range of candidate genes (Rieger and Chu, 2004), *CDKN1A*, *GADD45A*, *BBC3* and *CCNB1* were selected for the present work.

It has been established that there are significant correlations between SNPs (single nucleotide polymorphisms) in genes related to the biological response to radiation injury (e.g. genes involved in DNA repair (*BRCA1* and 2, *XRCC1*)) or DNA damage signalling (e.g. *ATM*) and the risk of radiation-induced normal tissue reaction (Andreassen *et al*, 2003). Very recently, it has been shown that chromosomal radiosensitivity can be influenced by genetic polymorphisms and that SNPs in nonhomologous end-joining genes may be associated with breast cancer risk (Willems *et al*, 2008). These genetic variations can affect gene transcription, the stability of the mRNA, the protein structure or protein–protein interactions. Of particular interest are the nonsynonymous SNPs that lead to amino-acid changes in the translated protein, therefore, having the potential to alter protein function and contribute to variations in response between patients. It has been suggested that genetic polymorphisms in *TP53* might affect some of its functions. Among the *TP53* variants, the common *TP53* Arg72Pro has been shown to differ biochemically and biologically leading to different levels of apoptotis (Thomas *et al*, 1999) and exerting different effects on cell cycle progression (Pim and Banks, 2004). *CDKN1A* is transcriptionally activated by *TP53* and both genes play a direct role in G1/S checkpoint control in response to IR. Earlier work suggested that there is a possible combined effect of polymorphisms in the two genes. An association between the risk of acute skin toxicity and *TP53* 72Pro carriers in those with the *CDKN1A* 31Ser genotype in a subset of normal weight patients treated with radiotherapy for breast cancer has been shown (Tan *et al*, 2006). Therefore, to examine the relationship between normal tissue radiosensitivity and polymorphisms in key genes, we also genotyped our samples for these two non-synonymous SNPs *TP53* codon 72 Arg/Pro G>C and *CDKN1A* codon 31 Ser/Arg C>A, previously associated with radiation sensitivity (Alsbeih *et al*, 2007).

## MATERIALS AND METHODS

### Samples

Lymphocyte samples, separated on Histopaque-1077 (Sigma Aldrich, Poole, Dorset, UK) and frozen within 24 h of blood collection from 22 sporadic breast cancer patients 30–72 years old treated at the Christie Hospital in Manchester, were obtained between 1993 and 1996 after local excision but before radiotherapy, in a prospective study of acute skin reactions (Barber *et al*, 2000). None of the patients had had a mastectomy or had received chemotherapy, but most of the patients involved in the study were taking tamoxifen. The previously published work demonstrated no significant relationship between chromosomal radiosensitivity, tamoxifen intake, menopausal status, age or smoking history (Barber *et al*, 2000). Consent was obtained from the patients and South Manchester Medical Research Ethics Committee approved the study. Radiotherapy was delivered with a tangent pair of fields at a prescribed dose of 40 Gy to the mid-plane. Measurements of skin reactions were made on 201 patients, of which 13 showed severe reactions (sufficient to warrant premature termination radiotherapy, or where most of the breast had moist desquamation), the remainder having normal reactions. The mean age of the patients was 52.6 (normal reactors (NRs)) and 53.6 (severe reactors (SRs)). Lymphocytes for all patients were screened for homozygous or heterozygous ATM mutations but none were detected (Appleby *et al*, 1997).

Lymphocytes separated from blood samples from two healthy female donors (PH4B and JM1) were used to produce disease-free control T-cell lines; in addition, an AT T-cell line (AT58) was used, originally obtained from Dr C Arlett, University of Sussex (Cole and Arlett, 1994).

### Cell growth

T-lymphocyte cultures were prepared using the method described previously. Briefly, frozen stocks were thawed and cultured in 10 ml of stimulating medium (SR10) at 2–3 × 10^5^ cells per ml. Cultures were incubated for about 4 days at 37°C, 5% CO_2_ atmosphere, then disaggregated and counted daily, maintaining cell density between 0.2 and 1 × 10^6^ cells per ml.

### Irradiation of lymphocyte cultures

Cells were seeded at a density of 4 × 10^5^ cells per ml in GR10 medium and irradiated with 2 Gy using a Siemens Stabilipan Therapy X-ray set (output 14 mA 250 kVp, 0.7 Gy min^−1^) at the MRC Harwell, then incubated in GR10 medium at 37°C and 5% CO_2_ for 2 h before processing.

### RNA extraction and reverse transcription

Cells (2 × 10^6^) were collected for each sample point by centrifugation, re-suspended in RNA Later (Ambion, Applied Biosystems, Foster City, CA, USA) and stored at −80°C. Total RNA was prepared by using RNAqueous^®^-4PCR (Ambion) kit. Reverse transcriptase reactions were performed with 1–1.5 *μ*g of total RNA (High Capacity cDNA Archive Kit, Applied Biosystems).

### Quantitative real-time PCR

Real-time PCR was performed using iQ5 thermocyclers (Bio-Rad, Hercules, CA, USA). All reactions including no-template controls were run in triplicate using primer and probe sets for target genes (Table 1). FAM, HEX and Texas Red were used as fluorochrome reporters for the hydrolysis probes analysed in multiplexed reactions.

Data were collected and analysed by iQ5 Detection System software. Gene target *C*_t_ (cycle threshold) values were normalised to a Hypoxanthine–Guanine phosphoribosyltransferase 1 human (HPRT1) internal control. *C*_t_ values were converted to transcript quantity using standard curves obtained by serial dilution of PCR-amplified DNA fragments of each gene or cDNA. The linear dynamic range of the standard curves covering five orders of magnitude (serial dilution from 4.10^−2^ to 1024.10^−7^) gave typical PCR efficiencies >90% for each gene with *R*^2^>0.95. Relative gene expression levels after irradiation were similarly obtained for comparison with preirradiation controls.

### DNA extraction, amplification, sequencing and data analysis

The selected SNPs and PCR primers utilised are listed in Table 1. DNA was extracted using QIAamp DNA Blood Midi Kit (Qiagen, Hamburg, Germany). Relevant segments of DNA were amplified by thermal cycling, directly sequenced using the DYEnamic ET Dye Terminator Cycle Sequencing Kit (GE Heathcare, Piscataway, NJ, USA) and run on a MegaBase 1000 sequencer (Applied Biosystems). Sequencing results were aligned to the corresponding reference sequence and SNPs were genotyped using SeqManII sequence analysis software (DNASTAR Inc., Madison, WI, USA).

Analysis of variance (ANOVA) was used to compare the means of gene expression values between normals and overreactors. When possible, a nested ANOVA method was used to calculate the variance between experiments and the variance between groups and to draw comparisons.

The association between severity of acute reactions to radiotherapy and SNPs genotype was determined from odds ratios, confidence intervals and significance level or, where not applicable, Fisher's Exact tests. Statistical analyses used Epi Info database and statistics software for public health professionals (Centers for Disease Control and Prevention, Atlanta, GA, USA).

## RESULTS

**Figure 1:**
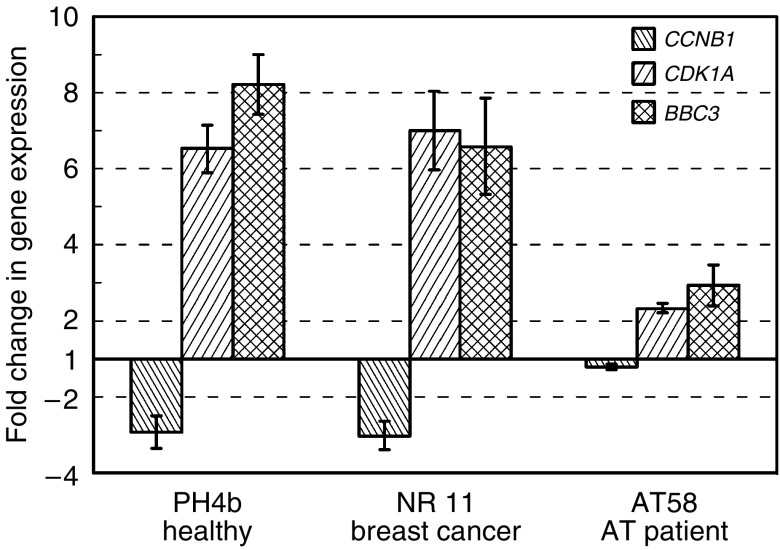
Quantitative PCR analysis of gene expression in cultured lymphocytes 2 h after 2 Gy X-rays from a healthy donor (PH4B), one breast cancer case with normal therapy reaction (NR 11) and an AT case (AT58). Values were normalised using *HPRT* as standard. The figures represent the ratio of level of expression after irradiation divided by the mock-treated cell expression levels. The mean value of 2–3 experiments reproduced at least twice each with three reactions are shown (error bars, s.e.).

To identify suitable genes, develop assays and establish their sensitivity, tests were carried out comparing responses of cultured T-lymphocytes from normal controls and an AT case. Cells from AT patients are defective in the initiation of cell-cycle checkpoints following DNA damage, as ATM kinase activity is critical for the appropriate initiation of signalling pathways. Results obtained for three genes *CCNB1*, *CDKN1A* and *BBC3* from a healthy control (PH4b), a breast cancer patient with normal therapy reaction (NR 11) and an AT (AT58) case are presented in Figure 1. Both the disease-free control PH4b and NR 11 have strong and similar radiation-induced decreases in cyclin B1 expression, 2.9- and 3-fold, respectively. AT58 cells showed only a marginal decrease of cyclin B1 mRNA of 1.2-fold. Both the cell-cycle arrest promoter *CDKN1A* and the proapoptotic *BBC3* in PH4b and NR 11 cells were strongly upregulated; AT58 responses were significantly less.

To examine the relationship between acute skin reactions and gene activation/repression after irradiation in *CCNB1*, *CDKN1*, *BBC3* and *GADD45A* (a well-characterised radiation-responsive gene; Grace *et al*, 2002), T-lymphocytes from 22 breast cancer patients were analysed. The NR and SR patient groups were comparable in size (11), sex, age and tumour status. Individuals showed a considerable range in baseline expression for the four genes (Table 2), although NR and SR groups were statistically indistinguishable.

**Figure 2:**
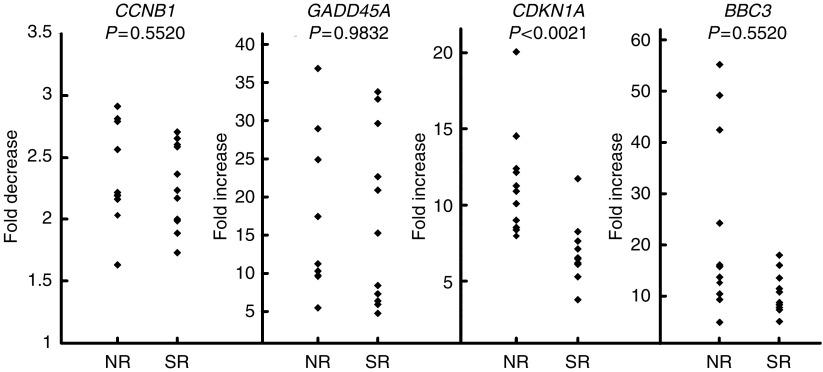
Gene expression analysis of *CCNB1*, *GADD45A*, *CDKN1A* and *BBC3* by quantitative PCR in 22 cultures of cultured T-lymphocytes from female breast cancer patients (11 SR and 11 NR). Values were standardised to *HPRT* expression level. The gene expression level ratio of irradiated/mock-treated cell is presented. The mean values of triplicate experiments, each with three reactions, are shown.

**Figure 3:**
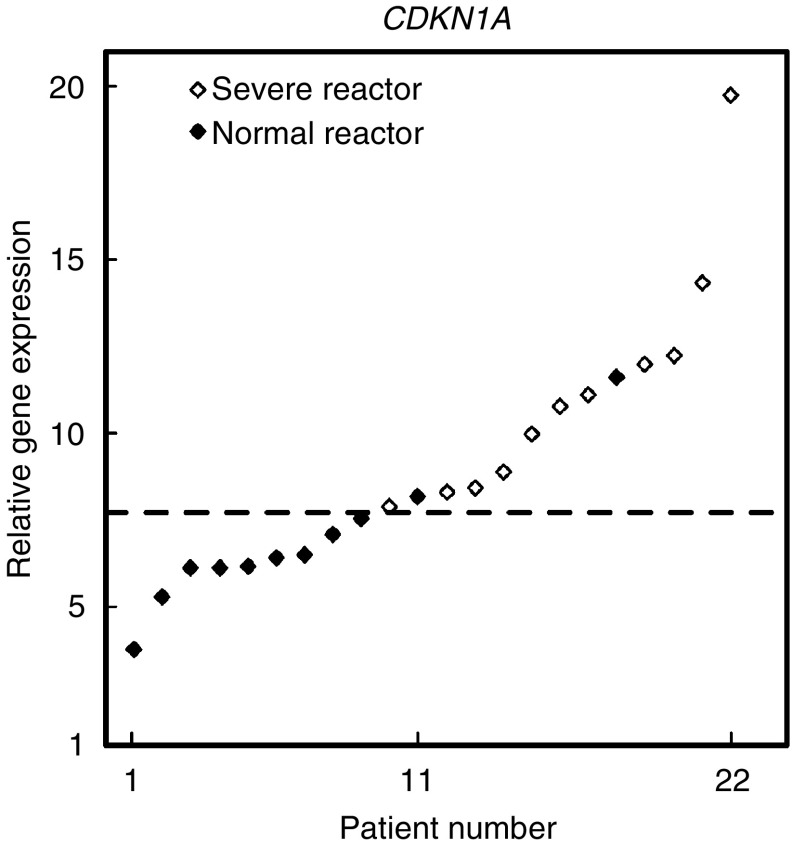
Gene expression analysis by quantitative PCR in 22 cultures of T-lymphocytes from female breast cancer patients (11 SR and 11 NR). The results presented show the cells response 2 h after 2 Gy X-rays and have been sorted into ascending magnitude of response (fold increase in *CDKN1A* gene expression). Values were standardised to *HPRT* expression level. The gene expression level ratio of irradiated/mock-treated cell is presented. The mean value of triplicate experiments, each with three reactions, are shown. The dotted line indicates the retrospectively defined cutoff for predicting radiation toxicity, with two false negatives and no false positive. Filled diamonds: SR, empty diamonds: NR

After irradiation, there was a wide range of responses in terms of either repression (*CCNB1*) or induction (*GADD45A*, *CDKN1A*, *BBC3*) as shown in Figure 2. Statistical analysis of results is shown in Table 2, no statistical differences were found between NR and SR responses for *GADD45A* (*P*=0.98), *CCNB1* (*P*=0.55) and *BBC3* (*P*=0.55). By contrast, radiation-induced increases in *CDKN1* expression were significantly less in SR than NR (*P*<0.0021). Nested ANOVA tests confirmed that replicate experiments performed consistently without significant differences between experiments (*P*=0.308). In Figure 3, the relative *CDKN1A* expression for all 22 patients is presented sorted in terms of increasing magnitude of response. There is an overlap between NR and SR and it is not possible to predict the normal tissue response on an individual basis for all samples. However, using a cutoff of seven-fold increase, reaction status in ∼91% (20/22) would be correctly identified with only two false negatives and no false positives.

To determine whether the *CDKN1* response differences between NR and SR could be related to polymorphisms in *TP53* codon 72 or *CDKN1A* codon 31, sequencing was performed on DNAs from lymphocyte cultures of 12 SR patients as described in Materials and Methods plus two additional SR cultures. No significant differences in frequency were found between groups for either polymorphism (Table 3).

## DISCUSSION

In this study, QRT-PCR assays have been used to assess the predictive value of four genes for acute skin reactions to radiotherapy. The transcripts levels of *CCNB1*, *CDKN1A*, *BBC3* and *GADD45*, all identified as radiation responsive in previous gene expression array studies (Tusher *et al*, 2001; Jen and Cheung, 2003) and recognised as biomarkers of radiation exposure, have been analysed. This study, therefore, addresses the hypothesis that expression differences in genes involved in the DDR network associate with severity of normal tissue radiation toxicity. Expression of these genes was analysed in a set of breast cancer patients with differences in sensitivity to radiation treatment.

Specific genetic variation among populations contributes appreciably to differences in gene expression phenotypes (Morley *et al*, 2004; Spielman *et al*, 2007). Gene expression signatures highlighting the molecular characteristics of individual patients are already useful for personalised cancer therapy (Garman *et al*, 2007). In this study, it was speculated that intrinsic radiosensitivity differences in most or all tissues in the body are reflected in the expression of genes influencing radiation sensitivity.

The DDR network senses genotoxic stress and coordinates a response, which includes activation of transcription, cell cycle checkpoints, apoptosis, senescence and DNA repair processes. This coordination is essential for cell survival.

Results comparing AT and normal cells are in good agreement with knowledge of the *ATM* DNA-damage-dependent signal transduction pathway. Unlike the Rieger *et al* (2004) study where microarray data on lymphoblastoid cells failed to predict toxicity in subjects with defined genetic defects like AT, the present results with *CCNB1*, *CDKN1A* and *BBC3* clearly demonstrated differential responses between a healthy donor and an AT case.

Of the genes studied, only *CDKN1A* expression allowed discrimination of NR from SR on average and in ∼91% of individuals. Although *CDKN1A* is a well-known damage response gene, it was not previously identified in array-based screens for genes predictive of early (Rieger *et al*, 2004) or late (Svensson *et al*, 2006) toxicity. *CDKN1A* is cyclin-dependent kinase inhibitor-1A also referred to as p21; this gene codes for a protein, which inhibits cyclin kinase activity; it is tightly regulated at the transcriptional level by p53 protein and serves as the effector of *TP53* cell cycle control. *CDKN1A* is well known as a biological indicator of IR exposure in humans (Amundson *et al*, 2003). It is, therefore, not entirely surprising that *CDKN1A* expression level may be predictive of radiation toxicity; a plausible biological explanation exists in that knockout mouse studies demonstrate *CDKN1A*, which plays a role in protecting intestinal epithelial cells from radiation-induced apoptosis (Wang *et al*, 1997). Loss of *CDKN1A* in a context of *ATM* deficiency also leads to accelerated kinetics of acute radiation toxicity *in vivo*, principally because of effects on the gut epithelium, suggesting that *CDKN1A* has a crucial role in acute normal tissue response to IR (Wang *et al*, 1997). Amundson *et al* (2004) showed that *in vivo* patterns of stress-gene induction by IR in blood are similar to those observed *ex vivo.* They demonstrated that *CDKN1A* is induced *in vivo* in humans undergoing total body irradiation and was one of the most promising biomarkers that also showed interindividual variation in response. The expression level of one gene in lymphocytes in response to radiation cannot alone explain the response of complex tissues; the vascular system or cytokines involved in the inflammation process must contribute as well. Nevertheless, our results support the concept that gene expression differences, perhaps reflecting underlying genetic variants, seem to be associated with radiation toxicity.

At least 40 polymorphisms of *CDKN1A* have been identified (Li *et al*, 2005) of which 35 are intronic. Only seven have an allele frequency >10%, but they are ∼1.3 kb upstream of exon 2. Among the common SNPs, *CDKN1A* C98A is found in exon 2 and causes a non-synonymous serine-to-arginine substitution at codon 31. This SNP is located in a highly conserved region of the gene (Chedid *et al*, 1994) and, as it is likely to change the properties of the protein, it is the most widely studied of the *CDKN1A* SNPs.

In our group of patients, *CDKN1A* gene expression neither associated with the *CDKN1A* C31A SNP nor with the common SNP in *TP53* (G72C). Larger studies would be needed to confirm these results. However, our results are in line with those obtained by Tan *et al* (2006) who examined the impact of these two SNPs on radiation response using the end point of acute skin toxicity after radiotherapy for breast cancer, no clear-cut association was found. In an *in vitro* study, Alsbeih *et al* (2007) described a significant association between cellular clonogenic radiosensitivity and *TP53* G72C but not *CDKN1A* C31A. The discrepancy between these two sets of data may be due to the presence *in vivo* of cofactors specific to each patient that could mask subtle SNP associations.

There are other polymorphic SNPs in *CDKN1A*, which could be more relevant to the rate of gene expression, for example, some SNPs in the first intron containing the promoter with the p53 binding site. These SNPs have wide range of frequency (∼0.001–0.48) in the population and could influence the rate of gene expression by modifying the binding affinity of transcription factors. Therefore, screening of the whole *CDKN1A* genetic region would be necessary to determine the influence of all these SNP variations on gene expression and toxicity to radiation therapy.

To summarise, the data presented here support the hypothesis that at least some breast cancer patients, who develop severe reactions to radiotherapy, have an intrinsic radiosensitivity that can be identified in peripheral blood lymphocytes by quantifying gene expression response to IR and that *CDKN1A* is a surrogate marker for early effects of radiation therapy. Confirmation and extension of these results may enable development of simple clinical tests to predict the likely level of radiation toxicity and to individualise patient treatment.

## Figures and Tables

**Table 1:** PCR primers and probes for quantitative analysis of gene expression and primers used for PCR amplification and DNA sequencing of SNPs

	**Database acc no.**		
**Genes**	**Genebank/dbSNP**	**PCR primers, Fwd, Rev**	**Probes**
*HPRT1*	NM_000194.1	TCAGGCAGTATAATCCAAAGATGGT	CGCAAGCTTGCTGGTGAAAAGGACCC
		AGTCTGGCTTATATCCAACACTTCG	
*CCNB1*	NM_031966.2	ATAAGGCGAAGATCAACATGGC	CGCAAAGCGCGTTCCTACGGCC
		TTTGTTACCAATGTCCCCAAGAG	
*CDKN1A*	NM_078467.1	GCAGACCAGCATGACAG	TTTCTACCACTCCAAACGCCGGCT
		TAGGGCTTCCTCTTGGA	
*GADD45A*	NM_001924.2	CTGCGAGAACGACATCAAC	ATCCTGCGCGTCAGCAACCCG
		AGCGTCGGTCTCCAAGAG	
*BBC3*	NM_014417.2	CGGAGACAAGAGGAGCAG	CCCTCACCCTGGAGGGTCCTGT
		GGAGTCCCATGATGAGATTG	
			
*SNPs*			
*CDKN1A*	rs1801270–codon 31	CGCCATGTCAGAACCGGCT	
	SNP C/A, Ser/Arg	TTCCATCGCTCACGGGCC	
*TP53*	rs1042522–codon 72	TGGTCCTCTGACTGCTCTTTT	
	SNP G/C, Arg/Pro	AACTGACCGTGCAAGTCACA	

**Table 2:** Expression of genes in NR and SR samples

	**Basal expression**			**Expression 2 h after 2 Gy x-irradiation**		
	**Mean (95% conf. interval)**			**Mean (95% conf. interval)**		
	**NR**	**SR**	*P*-value*	**NR**	**SR**	*P*-value*
*CCNB1*	2.859 (2.233–3.484)	3.001 (2.341–3.659)	0.7487	2.341 (2.153–2.529)	2.267 (2.093–3.441)	0.552
*CDKN1A*	0.748 (0.141–1.356)	0.722 (0.573–0.872)	0.9298	11.241 (8.607–13.874)	6.795 (5.5541–8.05)	0.0021^**^
*GADD45A*	0.052 (0.014–0.09)	0.088 (0.024–0.151)	0.3256	17.168 (8.961–25.374)	17.062 (9.436–24.688)	0.9832
*BBC3*	0.045 (0.02–0.069)	0.065 (0.042–0.087)	0.1991	23.045 (11.434–34.817)	10.585 (7.916–13.255)	0.552

Mean values represent the expression level of each gene normalised to the house keeping control HPRT and for irradiated samples, relative to unirradiated controls.

^*^ANOVA test for heterogeneity between NR and SR groups, *P*-values ^**^P<0.0021, significant.

**Table 3:** Genotype and allelic frequencies of two polymorphisms assessed in 25 breast cancer patients treated with radiotherapy, 11 normal reactors (NR) and 14 severe reactors (SR)

	**Radiosensitivity groups *n* (%)**		**Odd ratio**	
**Genotype and allele**	**Severe reactors *n*=14**	**Normal reactors *n*=11**	**(95% conf. interval)**	**Significance level *P***
*CDKN1A (codon 31 C>A Ser/Arg)*				
*Genotypes*				
C/C	10 (71)	8 (73)		
C/A	4 (29)	2 (18)	N/A	1.00^*^
A/A	0 (0)	1 (9)	N/A	0.47^*^
				
*Allelic frequencies*				
C	24 (86)	18 (82)		
A	4 (14)	4 (18)	N/A	0.72^*^
				
*TP53 (codon 72 G>C Arg/Pro)*				
*Genotypes*				
G/G	9 (64)	6 (55)		
G/C	3 (21)	3 (27)	N/A	1.00^*^
C/C	2 (14)	2 (18)	N/A	1.00^*^
				
*Allelic frequencies*				
G	21 (75)	15 (68)		
C	7 (25)	7 (32)	0.71 (0.17–2.92)	0.59

N/A=not applicable. ^*^Two-tailed Fisher's Exact test.
